# Detection of alteration in carotid artery volumetry using standard-of-care computed tomography surveillance scans following unilateral radiation therapy for early-stage tonsillar squamous cell carcinoma survivors: a cross-sectional internally-matched carotid isodose analysis

**DOI:** 10.1016/j.ctro.2025.100912

**Published:** 2025-01-06

**Authors:** Efstratios Koutroumpakis, Mohamed A. Naser, Abdallah Sherif Radwan Mohamed, Salman A. Eraj, Andrea Jarre, Jay C. Shiao, Mona Kamal, Subha Perni, Jack P. Phan, William H. Morrison, Steven J. Frank, G.Brandon Gunn, Adam S. Garden, Anita Deswal, Jun-ichi Abe, David I. Rosenthal, Elie Mouhayar, Clifton D. Fuller

**Affiliations:** a Department of Cardiology, Division of Internal Medicine, The University of Texas MD Anderson Cancer Center, 1515 Holcombe Blvd # 1451, Houston, TX 77030, USA; b Department of Radiation Oncology, The University of Texas MD Anderson Cancer Center, 1515 Holcombe Blvd, Houston, TX 77030, USA; c Department of Radiation Oncology, Baylor College of Medicine, Houston, TX, USA; d Department of Radiation Oncology, City of Hope Atlanta, Atlanta, GA, USA; e Department of Radiology, München Klinik Bogenhausen, Munich, DE, Germany; f Department of Radiation Oncology, The University of Kansas Cancer Center, Kansas City, KS, USA; g Department of Symptom Research, The University of Texas MD Anderson Cancer Center, Houston, TX, USA

**Keywords:** Radiation therapy, Radiation-induced carotid artery disease, Carotid artery volumetry, Computed tomography, Tonsillar cancer, Head and neck cancer

## Abstract

**Aim:**

This study leveraged standard-of-care CT scans of patients receiving unilateral radiotherapy (RT) for early tonsillar cancer to detect volumetric changes in the carotid arteries, and determine whether there is a dose–response relationship.

**Methods:**

Disease-free cancer survivors (>3 months since therapy and age > 18 years) treated with intensity modulated RT for early (T1-2, N0-2b) tonsillar cancer with pre- and post-therapy contrast-enhanced CT scans available were included. Patients treated with definitive surgery, bilateral RT, or additional RT before the post-RT CT scan were excluded. Isodose lines from treatment plans were projected onto both scans, facilitating the delineation of carotid artery subvolumes in 5 Gy increments (i.e. received 50–55 Gy, 55–60 Gy, etc.). The percent-change in sub-volumes across each dose range was examined.

**Results:**

Among 46 patients, 72 % received RT alone, 24 % induction chemotherapy followed by RT, and 4 % concurrent chemoradiation. The median interval from RT completion to the latest, post-RT CT scan was 43 months (IQR 32–57). A decrease in the volume of the irradiated carotid artery was observed in 78 % of patients, while there was a statistically significant difference in mean %-change (±SD) between the total irradiated and spared carotid volumes (−7.0 ± 9.0 vs. + 3.5 ± 7.2, respectively, p < 0.0001). Chemotherapy use, in addition to RT, was associated with a significant mean %-decrease in carotid artery volume compared to RT alone. No significant dose–response trend was observed in the carotid artery volume change within 5 Gy ranges.

**Conclusions:**

Our data show that standard-of-care oncologic surveillance CT scans can effectively detect reductions in carotid volume following RT for oropharyngeal cancer. Changes were equivalent between studied dose ranges, denoting no further dose–response effect beyond 50 Gy.

## Highlights

• Oncologic CT scans detected reductions in carotid artery volume post radiotherapy.
• Chemoradiation correlated with larger volume loss compared to radiation alone.
• No dose–response effect was detected in patients treated with > 50 Gray.
• Carotid volume changes for stroke risk stratification warrants further evaluation.

## Introduction

Oropharyngeal cancer (OPC), including tonsillar cancer, is the most common form of head and neck cancer, accounting for over 54,000 new cases in the US in 2023 alone [1]. The rising prevalence of HPV infection is leading to an increase in OPC in younger patients (age < 55), who have greater survival rates, with the majority living on for decades [1]. This increase in survival brings a greater focus to treatment-related side effects, with carotid artery stenosis (CAS) and resultant cerebrovascular accident (CVA), being among the most serious ones. The relative risk of transient ischemic attack (TIA) or CVA is at least doubled by head and neck radiotherapy (RT), the backbone of OPC treatment [2]. Despite the relatively high incidence of TIA and CVA among patients treated with neck RT (approximately 5 % in 5 years and 10 % in 10 years), evidence-based guidelines for the management of radiation-induced carotid artery disease (RICAD) are largely anecdotal [3], [4], [5], [6]. Currently, a major obstacle to the development of such guidelines is the lack of a quantified dose-risk relationship. Historically, carotid arteries received a uniform RT dose; however, since the early 2000′s, RT techniques, such as intensity-modulated RT (IMRT) have delivered a variable, non-homogenous dose to the carotid arteries, making the already crude estimators of CAS so anachronistic as to be functionally non-usable for risk stratification. Furthermore, several imaging modalities such as carotid ultrasound, computed-tomography (CT) scans and magnetic resonance imaging have been utilized to detect structural changes that predict subsequent development of clinical CAS. However, there is no evidence-based guidance as to which patients will benefit by which imaging, resulting in a heterogeneous use of these modalities in clinical practice. Development of personalized surveillance programs for the detection of RICAD based on baseline clinical and imaging characteristics as well as RT dose characteristics remains an unmet need. Standard-of-care CT scans with contrast, optimized for soft tissue visualization for staging, are performed around the time of diagnosis of OPC as well as within 6 months of treatment conclusion for determination of response [1]. Furthermore, longer term follow up CT scans of the head and neck area are frequently performed in OPC survivors to detect cancer recurrence or therapy-related complications. While the soft-tissue visualization optimized CT scans with contrast generally available for tonsillar cancer patients are not ideal for study of the carotid arteries, sufficient resolution is still present to accurately define the vessel’s intraluminal diameter from the CCA’s origin at the aorta to the ICA’s entry of the skull as compared to ultrasound (US).

Previous studies have reported that RT to the neck leads to increases in the intima media thickness (IMT) of the carotid arteries and decreases in their intraluminal area/volume [7]. Common carotid artery IMT has been shown to predict development of TIA/CVA independently of traditional cardiovascular risk factors [8]. However, common carotid artery IMT only assesses focal changes and does not capture the whole extend of radiation-induced carotid artery injury, which is a more diffuse process compared to carotid atherosclerosis in the general population [9]. Therefore, we propose the use of widely available standard-of-care oncology CT scans to assess extracranial carotid artery intraluminal volume changes as an indicator of the full extent of radiation-induced carotid artery injury and a measure that can assist in identifying patients at risk of CAS in addition to standard practice.

In previous work, Carpenter et al. performed an assessment of US-detected carotid stenosis (defined as ≥ 50 % luminal reduction on imaging) in 366 patients with prior RT. Using these criteria, no formal dose–response correlation could be defined [10]. However, US is limited by the lack of volumetric imaging across the entire carotid volume. Consequently, in a larger longitudinal series, Carpenter et al. recently demonstrated that nearly all dose-levels between 10–70 Gy were associated with CAS risk increase, echoing prior work by van Aken, which showed that in unscreened patients a bilateral volume receiving ≥ 10 Gy (V10) was associated with ischemic stroke risk [11], [12].

While substantive vascular disease (asymptomatic carotid stenosis > 50 %) and consequential (ischemic) late events have been demonstrated to have an association with low dose, we hypothesized, based on related prospective US assessment of unilateral carotids, that early (<5-year) volumetric changes could be potentially detectable using volumetric imaging, without referral for dedicated CT-or MRI-angiography. The specific aims of the current study included: 1) Demonstration of the technical feasibility of standard-of-care CT head and neck scans to detect alteration in post-RT carotid volumes. 2) Evaluation of dose–response of local carotid injury (e.g. at the location of dose) through isodose mapping. 3) Generation of preliminary data for future prospective observational and interventional approaches to early carotid radioinjury. Identifying vulnerable patients for CAS, utilizing standard-of-care surveillance CT scans and defining radiation-dose–response parameters associated with carotid injury would enable development of improved treatment guidelines and actionable dose-modification strategies that will lead to decreased radiation-associated TIA/CVAs, thereby reducing therapy-attributable morbidity and mortality and improving patient quality of life.

Methods:

### Patient population and data collection

Disease-free cancer survivors (18 years or older at diagnosis) previously treated for oropharyngeal squamous cell carcinoma at the University of Texas M.D. Anderson Cancer Center from October 2016 to May 2017 were identified. We utilized an extant dataset of CT images and clinical measures collected under an IRB-approved retrospective chart review protocol. Patients who were treated with unilateral intensity modulated RT (IMRT for early (T1-2, N0-2b) tonsillar cancer were included. We elected to study patients with early tonsillar cancer since they have low-probability of cancer-specific mortality and because early tonsillar cancer is often amenable to unilateral RT. With both a treated and untreated artery, the untreated carotid artery serves as a control to account for carotid artery volume changes related to patient-specific atherosclerotic processes. By leveraging intra-patient matched carotid comparisons, the variability associated with inter-patient atherosclerotic, cardiovascular, genetic or environmental confounders is modulated more effectively than with case-control or propensity matched cohorts. Patients treated with definitive surgery, bilateral RT, or additional RT prior to post-RT CT scan were excluded. Patient demographic, tumor and treatment characteristics were collected [13].

### Computed tomography scans

**Fig. 1:**
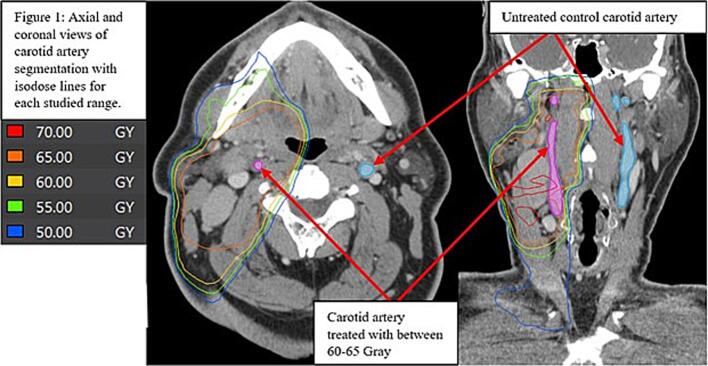
Axial and coronal views of carotid artery segmentation with isodose lines for each studies range.

Pre-RT CT scans included contrast-enhanced CT scans of the neck prior to initiation of RT. Post-RT scans included the last available (most recent) contrast-enhanced CT scans of neck. Two CT scans (pre- and post-RT) were analyzed for each patient. Contrast-enhanced CT scans were exported from the institution PACS to VelocityAI (version 3.0.1) in DICOM format. The RT plan must have been available and was exported from Pinnacle^3^ (version 14.0) to VelocityAI. The ICA/CCA (internal carotid artery/common carotid artery) were manually segmented in VelocityAI from the base of the skull to its origin at the aorta as depicted in Fig. 1. We employed image registration in VelocityAI to align the planning CT with the pre- and post-therapy contrast-enhanced CT images. This alignment facilitated the transfer of isodose lines from the treatment plan to the pre- and post-therapy CT images. Consequently, we could delineate subvolumes of the carotid artery that received incremental dose ranges of 5 Gy, varying from 50 to 70 + Gy, as illustrated in Fig. 1.

### Statistical analysis

Descriptive statistics were used to summarize the patient, treatment, and tumor characteristics. A linear regression analysis was performed to evaluate the association of demographic and clinical characteristics with the mean percent change in irradiated carotid artery volume. The percent change in volume for each 5-Gy subvolume was calculated and compared to the contralateral side by the Wilcoxon rank sum test. Analysis of variance (ANOVA) was conducted in regards to the 5 Gy ranges and percent volume changes as continuous variables, in addition to literature-reported correlates of CAS (e.g. systemic therapy, neck dissection status, smoking status).

## Results

A total of 46 patients were included in this analysis. Mean age ± SD was 54.5 ± 9.1 years, while 30 % of patients were women and 96 % white. Forty-three % of patients were either active (13 %) or former (30 %) smokers. None of the patients had CAS (>50 % stenosis) at baseline. Most patients who were tested had human papilloma-associated (HPV + ) disease (25/27; 93 %). In terms of cancer therapy, 72 % received RT alone, 24 % received induction chemotherapy followed by RT, and 4 % received concurrent chemoradiation. Neck dissection was performed in 20 % of patients. Most patients received 66–69 Gy of RT dose (87 %) divided in 30 or more fractions. Table 1 includes additional demographic and clinical characteristics.

**Table 1:** Demographic and basic clinical characteristics of 47 patients with early-stage tonsillar squamous cell carcinoma treated with unilateral neck radiation therapy.

	Total N = 46
Age, mean in years (SD)	54.5 (9.1)
Female sex, N (%)	14 (30.4)
White race, N (%)	44 (95.6)
Hispanic ethnicity, N (%)	2 (4.3)
Smoking history, N (%) Active Former	20 (43.4) 6 (13.0) 14 (30.4)
HPV infection, N (%)	25/27 (92.5)
Cancer Stage, N (%) T1N0 T1N1-2b T2N0 T2N1-2b	3 (6.5) 29 (63.0) 3 (6.5) 11(23.9)
Cancer therapy, N (%) RT alone Induction chemotherapy + RT Concurrent chemotherapy + RT Neck dissection	33 (71.7) 11 (23.9) 2 (4.3) 9 (19.6)
RT Fractions, N (%) 30 33 35 40	40 (87.0) 3 (6.5) 1 (2.2) 2 (4.3)
RT dose delivered, N (%) 60–64 65–69 70+	2 (4.3) 40 (87.0) 4 (8.7)

The median time from RT completion to the latest available, post-RT CT was 43 months (IQR 32–57). Irradiated carotid shrinkage was observed in 78 % and there was a statistically significant difference in the mean percent change (±SD) of the total irradiated versus spared carotid artery volumes, −7.0 ± 9.0 vs. + 3.5 ± 7.2, respectively, (p < 0.0001).

**Fig. 2:**
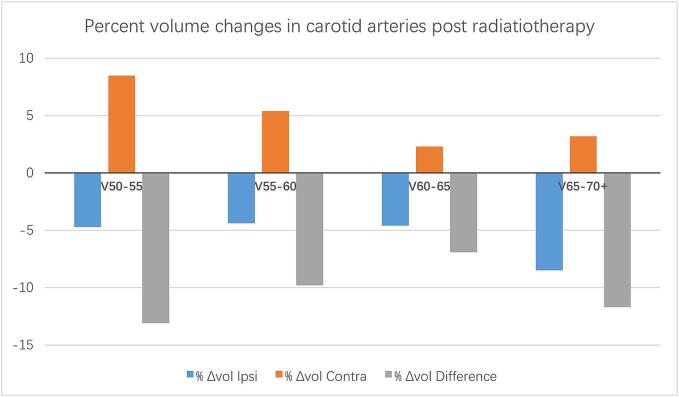
Mean percent volume change before and after radiation therapy in the ipsilateral (blue) and contralateral sides (orange) as well as mean difference in percent volume change (gray) in association with radiation dose volumetric parameters. (For interpretation of the references to colour in this figure legend, the reader is referred to the web version of this article.)

The mean percentage changes in subvolumes of the carotid artery that was exposed to 50–55, 55–60, 60–65 and 75–70 + Gy in comparison to the mean percentages changes in the subvolumes of the contralateral carotid artery are presented in Table 2. No significant trend (dose–response relationship) was found on analysis of 5 Gy range subvolumes via one-way ANOVA, the differences in mean percent changes (±SD) for the 50–55, 55–60, 60–65, and 65–70 + Gy range subvolumes (irradiated minus spared) being −13.1 ± 14.7, −9.8 ± 14.9, −6.9 ± 16.2, −11.7 ± 11.1, respectively (p = 0.217; Table 2 and Fig. 2). Two patients in cohort (4 %) developed a CVA during the follow up.

**Table 2:** Mean percent volume change before and after radiation therapy in the ipsilateral and contralateral sides as well as mean difference in percent volume change (ipsilateral minus contralateral) in association with radiation dose volumetric parameters.

Subvolumes	Mean % Volume change (pre- and post-RT) in ipsilateral carotid artery (SD)	Mean % Volume change (pre- and post-RT) in contralateral carotid artery (SD)	Mean difference in % volume change (ipsilateral – contralateral) (SD)
V50-55 Gy	−4.7 (11.7)	8.5 (17.5)	−13.1 (14.7)
V55-60 Gy	−4.4 (14.9)	5.4 (16.5)	−9.8 (14.9)
V60-65 Gy	−4.6 (14.4)	2.3 (12.8)	−6.9 (16.2)
V65-70 + Gy	−8.5 (10.6)	3.2 (8.2)	−11.7 (11.1)

No significant difference in overall carotid artery (ICA/CCA) volume between the treated and untreated sides were detected among patients who received systemic therapy versus those who did not (p > 0.05). All patients who received chemotherapy, were treated with 66 Gy of RT, while the mean dose of RT ± SD among patients treated with RT alone was 66.48 ± 1.80 (p = 0.34). Analysis by both receipt of systemic therapy and by 5 Gy range subvolumes via one-way ANOVA similarly did not reveal any statistically significant difference. There was not a statistically significant difference in the total carotid artery (ICA/CCA) volume change between current or former smokers versus nonsmokers, nor was there a significant difference when analyzing additionally by 5 Gy range subvolumes via one-way ANOVA. Finally, neck dissection was not associated with a statistically significant difference in overall carotid artery (ICA/CCA) volume via Wilcoxon test (p > 0.05), nor was there a significant difference via one-way ANOVA on analysis by 5 Gy range subvolumes (p > 0.5).

In a linear regression analysis evaluating the association of demographic and clinical characteristics with the mean % volume change in the irradiated carotid arteries, only systemic chemotherapy was significantly associated with a volume decrease (Table 3). Age, sex, race, smoking history, HPV infection, neck dissection, RT dose and RT fractions were not associated with significant carotid volume changes.

**Table 3:** Linear regression analysis evaluating the association of demographic and clinical characteristics with mean % volume change in the carotid arteries exposed to radiation therapy.

	Estimated coefficients	95 % Confidence intervals	p-value
Age, mean	0.002	(−8.97e-4)-0.005	0.169
Female sex	0.035	(−0.023)-0.092	0.235
White race	−0.078	(−0.208)-0.053	0.236
Smoking history (active or former)	−0.011	(−0.065)-0.043	0.677
HPV infection	0.059	(−0.062)-0.179	0.327
T stage (T2-T1)	0.016	(−0.043)-0.075	0.582
Lymph node involvement	−0.053	(−0.132)-0.025	0.178
Neck dissection	0.021	(−0.034)-0.075	0.450
Chemotherapy	−0.072	(−0.128)-(−0.016)	0.013
RT Fractions	0.005	(−0.007)-0.0173	0.391
RT dose delivered	0.005	(−0.013)-0.023	0.604

HPV: uman papilloma virus; RT: radiation therapy.

## Discussion

The current study demonstrated that standard-of-care CT scans can be used to detect carotid artery volume changes following unilateral RT for early tonsillar SCC. We found that patients treated with greater than 50 Gy to the majority of the carotid artery had a significant reduction in total carotid artery volume (ICA/CCA). Chemotherapy use, in addition to RT, was associated with a significant mean % carotid volume reduction compared to RT alone. On examination for a dose–response relationship by 5 Gy ranges from 50 to 55, 55–60, 60–65, 65–70 + Gy, no significant differences were found, suggesting no further dose–response effect beyond 50 Gy. Although there may be a dose–response threshold under 50 Gy, we were not able to investigate that question in our dataset as very small, discontinuous volumes of the carotid received these lower doses.

CAS following RT is well-documented. Based on a systematic review of the literature, the prevalence of CAS was increased by 16–55 % after RT; however the majority of studies were suboptimally matched, retrospective, and/or used historical radiation approaches [14]. Despite its prevalence, the pathogenesis of RICAD is still not completely understood [15], [16]. RICAD develops over years through typical atherosclerotic processes, but at a faster pace compared with unirradiated atherosclerotic arteries [17]. The interval from RT to symptomatic vascular changes ranges between several months to twenty years^2,32,3^ [18], [19]. For non-radiation related CAS, the most common sites are the carotid bifurcation and proximal segment of the internal carotid artery (ICA). This differs in RICAD, with the common carotid artery (CCA) being the most affected [9], [19]. RICAD sometimes extends beyond the radiation field, often to the proximal CCA and distal ICA, which are difficult to detect with US, but can be detected with CT [19], [20].

Current routine methods for imaging the carotid arteries for determination of stenosis include duplex sonography, CT angiography (CTA), digital subtraction angiography, and magnetic resonance angiography [21]. Traditionally, measurement of the lumen has been the standard method of determining CAS on angiographic imaging studies, but also measurement of artery thickness, such as intima-media thickness (IMT) and the interadventitial diameter (IAD) have emerged as avenues for detection of subclinical atherosclerosis, as well as prognostic tools [4], [22]. IMT, which is determined by ultrasound, has been shown to be an early marker of radiation-induced carotid artery damage and has been utilized by the majority of studies examining RICAD [15], [19], [23]. IAD and whole vessel diameter enlargement, which can be measured on CTA, have been found to occur early in atherosclerosis, become exaggerated in the presence of vulnerable plaques, and were correlated to cardiac events and CVA [22], [24], [25], [26]. IMT and IAD only assess local changes and might not capture the whole extend of RICAD. Carotid artery volume estimation by CT is a 3-dimentional measure that includes the whole length of the extracranial carotid artery and can detect subsegmental changes especially in the setting of heterogeneous dose delivery patterns of RT. Based on the results of the current study we propose the use of carotid artery intraluminal volumes by CT as a novel measure of radiation induced changes. Whether decrease in carotid artery volumes post radiation predicts the development of TIA/CVA needs to be further evaluated.

Patients with OPC undergo evaluation with several CT scans of the head and neck area with contrast for cancer diagnosis and staging purposes, determination of therapy response or evaluation for cancer or cancer therapy related complications [27]. While the soft-tissue visualization optimized CT scans generally available for tonsillar cancer patients are not optimized for study of the carotid arteries, sufficient resolution is still present to accurately define the external diameter, as well as identify plaques. CT with contrast also allows for good resolution of the entire vascular tree from the CCA’s origin at the aorta to the ICA’s entry of the skull as compared to ultrasound [4]. In this study, we proved that standard-of-care oncologic surveillance soft-tissue visualization optimized CT scans with contrast can be used to successfully monitor whole carotid artery temporal volume changes in association with RT dose exposure. This approach enables the potential (re)use of clinically collected imaging studies to identify vulnerable patients at risk of CAS and its devastating associated morbidity and mortality. Additionally, in our cohort, three out of four patients had a decrease in their carotid artery volume, while an average increase in the volume of the spared carotid artery was noted. We hypothesize that the irradiated carotid artery volume loss correlates with the soft-tissue neck fibrosis seen post RT and the increase in the spared carotid artery volume might be compensatory to the decrease in the volume of the irradiated artery in order to maintain unchanged intracranial blood perfusion. This hypothesis needs to be further tested in future studies.

When we evaluated the association of demographic and clinical characteristics with carotid artery volume changes, only chemotherapy use was significantly associated with significant carotid volume loss. This finding suggests that chemotherapy use potentiates the toxic effects of RT in the vascular system and is in line with previous literature [11], [28]. Age, smoking history, HPV infection, and neck dissection were not significantly associated with carotid artery volume loss in our cohort but this might be due to our small sample size. Furthermore, RT dose was not significantly associated with carotid volume loss either, which may be due to the fact that all patients were treated with > 50 Gray.

Two recent retrospective studies examined the relationship of radiation dose and risk of CAS (dose–response relationship) and reported increased risk with all dose-levels between 10–70 Gy [11], [12]. In our study, we did not find any significant difference in the percent volume change of the carotid artery in association with radiation dose for doses above 50 Gy divided in 5 Gy intervals. Lower doses could not be investigated as only small, discontinuous volumes of the carotids received doses < 50 Gy. It is possible that there is a dose–response threshold under 50 Gy, as the current literature indicates, above which there is a significant increase in the risk of CAS, but with no further increase in the risk for dose groups above 50 Gy. If indeed the dose–response threshold is as low as 10 Gy, that means that efforts to decrease RT dose for the treatment of OPC might not necessarily lead to a decrease in the incidence of RICAD, since RT dose in such low levels is ineffective.

Importantly, the lack of dose–response at therapeutic elective doses (i.e. > 50 Gy) suggests that for the foreseeable future, despite recent dose de-escalation applications (e.g. ECOG 3311 which prescribed minimum *50 Gy* to radiated post-surgical cases) [29], the majority of head and neck RT patients are at risk for carotid artery injury at levels detected in the current study. Moreover, the epidemic of radiocurable HPV + diseases, wherein the majority of patients can expect extended survival, often decades after therapy, suggest an unmet need for more careful surveillance of sub-clinical-to-chronic-to-symptomatic radiovascular toxicity. Consequently, additional efforts are needed to characterize and monitor whether the observed early changes in carotid lumen are indicative of later atherosclerotic disease states (e.g. asymptomatic carotid artery stenosis, plaque formation, TIA, CVA), and could serve as an early harbinger risk or as a risk stratification tool, easily integrated into standard CT surveillance imaging. Additionally, our data suggest that efforts to reduce carotid dose (e.g. unilateral radiotherapy [30], [31], margin reduction [32], or conformal techniques [33], [34], are justifiable based on secondary imaging showing sub-acute vascular injury, and should be consistently considered when feasible if they do not compromise tumor control or oncologic recurrence risk.

In terms of the incidence rate of TIA/CVA, previous data has suggested that it measures approximately 5 % in 5 years and 10 % in 10 years following RT [11]. Our results demonstrated an incidence rate of 4 % over a median follow up of 43 months, which is in line with the incidence rates reported in the literature.

This study has several limitations, including the small sample size and retrospective nature with its inherent risk of bias. Furthermore, most patients in this study were men and white, which decreases generalizability of our findings. Also, the median follow up of our study of 43 months is relatively short considering the late development of RICAD. Despite the small sample size and short follow up, we were still able to detect statistically significant changes in the main outcome reported in our study. Finally, our study does not include data on lipid levels and how lipid-lowering therapies such as statins would modify the change in carotid artery volumes.

## Conclusions

This study provides evidence that the volume of carotid artery significantly decreases following RT compared to the contralateral unirradiated carotid artery among patients with tonsillar cancer. Standard-of-care CT scans can be used to detect these changes and potentially help risk stratify patients who will benefit from dedicated carotid artery imaging and frequent surveillance. Based on these findings, future studies could enable use of standard surveillance CT scans to screen for carotid artery stenosis in head and neck cancer survivors as well as lead to the creation of clinically meaningful mechanisms to modify the risk of carotid radiation vasculopathy and reduce radiation-associated morbidity and mortality.

## Fundings

None.

## CRediT authorship contribution statement

**Efstratios Koutroumpakis:** Conceptualization, Formal analysis, Visualization, Writing – original draft. **Mohamed A. Naser:** Conceptualization, Data curation, Resources, Visualization, Writing – review & editing. **Abdallah Sherif Radwan Mohamed:** Data curation, Visualization, Writing – review & editing. **Salman A. Eraj:** Data curation, Visualization, Writing – review & editing. **Andrea Jarre:** Data curation, Visualization, Writing – review & editing. **Jay C. Shiao:** Data curation, Visualization, Writing – review & editing. **Mona Kamal:** Data curation, Visualization, Writing – review & editing. **Subha Perni:** Data curation, Visualization, Writing – review & editing. **Jack P. Phan:** Visualization, Writing – review & editing. **William H. Morrison:** Visualization, Writing – review & editing. **Steven J. Frank:** Visualization, Writing – review & editing. **G.Brandon Gunn:** Visualization, Writing – review & editing. **Adam S. Garden:** Visualization, Writing – review & editing. **Anita Deswal:** Resources, Visualization, Writing – review & editing. **Jun-ichi Abe:** Visualization, Writing – review & editing. **David I. Rosenthal:** Conceptualization, Project administration, Resources, Visualization, Writing – review & editing. **Elie Mouhayar:** Conceptualization, Project administration, Resources, Visualization, Writing – review & editing. **Clifton D. Fuller:** Conceptualization, Formal analysis, Project administration, Resources, Visualization, Writing – review & editing.

## Declaration of Competing Interest

The authors declare the following financial interests/personal relationships which may be considered as potential competing interests: EK is supported in part by NIH/NCI 1R01 HL157273 and CPRIT RP200381 both of which are not related to the current work. AD is supported in part by NIH and the Cancer Prevention Research Institute, has received consulting fees from Bayer, honorarium from Kaplan for Projects in Knowledge and travel support as speaker by the American College of Cardiology. EM has received consulting fees from RYVU and honoraria from UPTODATE as topic reviewer. CDF receives related grant, salary, and infrastructure support from MD Anderson Cancer Center via: the Charles and Daneen Stiefel Center for Head and Neck Cancer Oropharyngeal Cancer Research Program; the Program in Image-guided Cancer Therapy; and the NIH/NCI Cancer Center Support Grant (CCSG) Image-Driven Biology and Therapy (IDBT) Program (P30CA016672). Dr. Fuller has received unrelated grants/honoraria from Elekta AB, has received travel support from Philips Medical Systems, and has served in an advisory capacity for Siemens Healthineers.
